# Reflections on the Future of Pharmaceutical Public-Private Partnerships: From Input to Impact

**DOI:** 10.1007/s11095-017-2192-5

**Published:** 2017-06-06

**Authors:** Remco L. A. de Vrueh, Daan J. A. Crommelin

**Affiliations:** 1 Lygature, Utrecht, The Netherlands; 2 Department of Pharmaceutics, Utrecht Institute for Pharmaceutical Sciences, UIPS, Utrecht University, Utrecht, The Netherlands

**Keywords:** key drivers, performance evaluation, public-private-partnerships, R&D business models

## Abstract

Public Private Partnerships (PPPs) are multiple stakeholder partnerships designed to improve research efficacy. We focus on PPPs in the biomedical/pharmaceutical field, which emerged as a logical result of the open innovation model. Originally, a typical PPP was based on an academic and an industrial pillar, with governmental or other third party funding as an incentive. Over time, other players joined in, often health foundations, patient organizations, and regulatory scientists. This review discusses reasons for initiating a PPP, focusing on precompetitive research. It looks at typical expectations and challenges when starting such an endeavor, the characteristics of PPPs, and approaches to assessing the success of the concept. Finally, four case studies are presented, of PPPs differing in size, geographical spread, and research focus.

## Introduction

Bilateral R&D interactions between academic and industrial scientists have a long tradition. In the last decade, alongside these bilateral (‘vertical’) interactions, the multiple stakeholder partnerships called Public-Private Partnerships (PPPs) have emerged. These are R&D networks that do not work on a one-to-one basis but rather involve a range of stakeholders. As well as traditional academia and industry stakeholders, such a PPP may include charities, patient organizations, and even national competent authorities (‘regulators’).

The scope of this review is a discussion of the role and added-value of PPPs in facilitating *precompetitive multi-stakeholder collaborative research*. First, we will focus on the key drivers for different stakeholders as they embrace the multi-stakeholder collaborative research model. Second, we will provide insight into the challenges of managing multi-stakeholder collaborative research projects and how different PPPs have dealt with these challenges. Third, we will elaborate more on current models for monitoring and evaluating the performance of precompetitive PPPs. Finally, given the growing number of PPPs, we conclude by discussing their value for society and the need to justify the public and private investments being made (1,2).

## Shift in the Pharmaceutical Business Model: From Fully Integrated to Open Innovation

The process of bringing new treatments from “bench to bedside” has been described as a translation continuum, where various types of resources and areas of knowledge are involved when moving from basic fundamental research to proven clinical application and, eventually, medical practice (and back) (3). Major advances in a.o. molecular biology, functional genomics, and genetics have resulted in enhancing our scientific understanding of disease pathophysiology (4). Furthermore, the different ‘omics’ fields have enabled large-scale measurements of biological processes (5). Around the turn of the century, the general expectation by the global biomedical community, both public and private, was that this increased knowledge base would fuel the development of a new generation of innovative therapies, impacting public health (5). Despite the promise, these major advances in science have not yet increased the success rate of moving a compound from lab bench to first-in- man to approval and into medical practice (6,7). In fact, the rate of development by pharmaceutical companies of innovative medicinal products which were approved by regulatory authorities slowed down (8,9), indicating that a number of chasms continue to exist in the translational process. One explanation for the high rate of attrition in drug development mentioned in literature is that industry is increasingly targeting more complex diseases. Another is that regulatory authorities have become increasingly demanding (10–12).

The pharmaceutical industry is already confronted with the patent expiry of many blockbuster drugs, the so-called ‘patent cliff’ (13). The disappointing rate of development attrition has been a strong motivator, especially for large companies, to thoroughly review drug development strategies and accompanying processes (14). Pharma companies know that if the trend remains unaltered, costs of developing a drug will continue to increase and ultimately render the drug development process untenable (15). This would jeopardize the long-term profitability that allows them to reinvest in future R&D. As a first response, a wave of mergers and acquisitions has taken place to save costs and to counter the weakening pipeline. However, several studies have shown that these mergers and acquisitions have not resulted in improvement of R&D productivity (9,16,17). What seems to have been a more effective “productivity improvement” strategy has been the increased focus of the pharmaceutical sector on developing medicinal products for rare diseases, so-called orphan drugs (18). Recent 10-year approval/positive opinion overviews presented by the FDA reveal an upward trend in the number of drug approvals/positive opinions since 2010 (19). In 2015, almost half of the approvals/positive opinions in the US were for orphan drugs (20), compared with one third in 2005 (21). A similar trend is observed for Europe (22).

Alongside an increased focus on rare diseases, the role of large pharmaceutical companies within the field of pharmaceutical innovation has been changing over the past 2–3 decades (23). Companies continue to move away from the traditional “*fully integrated discovery and development company*” model, towards the adoption and implementation of various open innovation strategies such as strategic alliances, purchase of scientific services and in-licensing (24,25).

In the various models, companies tend to focus internally more on late (phase II/III) clinical development and distribution of products. At the same time, the open innovation models allow them to access efficiently the best science (26,27). Consequently, the discovery of potentially new therapies and subsequent pre-clinical and early (phase I/IIa) clinical evaluation have increasingly become the domain of academic parties and small and medium-sized enterprises (SMEs). Ultimately, the translation of basic scientific research into a potential treatment modality is then driven by “*fully integrated discovery and development networks*”, in which expertise and knowledge from academic parties, large pharmaceutical companies, SMEs, and other stakeholders converge in partnerships (25,28,29). As exemplified in Table 1, there is increasing focus on better understanding open innovation models in the bio-pharmaceutical sector and their impact on R&D productivity. This paper specifically focuses on the emergence of PPPs and their socio-economic impact.

**Table 1:** Examples of Research Focusing on Better Understanding the Open Innovation Concept in The Bio-pharmaceutical Sector

Article	Authors	Concise description
Organizational modes for Open Innovation in the bio-pharmaceutical industry: An exploratory analysis	Bianchi *et al*. (24)	•Based on two rounds of interviews with 20 industry experts, a model describing the adoption of inbound and outbound open Innovation by bio-pharmaceutical companies was presented •Bio-pharmaceutical firms employ a mix of open innovation modes (i.e. licensing agreements, alliances and supply/provision of technical and scientific services) to engage with large pharmaceutical companies, biotech firms and universities with the aim to acquire or commercially exploit technologies and knowledge.
Measuring Open Innovation in the Bio-Pharmaceutical Industry	Michelino *et al*. (30)	•Focus on degree of openness of 126 global top R&D spending companies (Period 2008–2012) •Model built from three different perspectives: inbound versus outbound processes, economic versus financial transactions and the nature of the traded entities: research and development, intellectual property and know-how •Negative correlation of openness degree with firm age, dimension and efficiency, with biotech companies being more open than pharmaceutical ones.
Models for open innovation in the pharmaceutical industry	Schuhmacher *et al*. (31)	•Based on an analysis of their R&D models, 13 multinational pharmaceutical companies were categorized with respect to their preference in innovation management (introverted or extroverted) and proportion of externally acquired R&D projects (low or high). •Based on the analysis and categorization, four types of open innovators were proposed: ‘knowledge creator’, ‘knowledge integrator’, ‘knowledge translator’ and ‘knowledge leverager’

## University-Industry Collaboration to Spur Biomedical R&D

Collaboration in R&D between academia and the pharmaceutical industry is not itself a novel concept. It has been around for a while, with the aim of sustaining pharmaceutical innovation and advancing new product development (32,33). The US is a prime example, where the Bayh-Dole Act of 1980 is considered an important spur for numerous collaborations between academic research groups and small biotech companies or large pharmaceutical companies (34,35). This development is part of a shift that universities have made, especially in the US and the EU, through which they consider technology transfer and commercialization as an integral part of their mission, a trend dubbed by D’Este and Perkman as the “Entrepreneurial University” (36).

University-industry collaboration within biomedical R&D over the last decades has moved beyond vertical, bilateral arrangements (37) such as contract research, consulting, and research agreements with an option to license drug candidates and technologies. It has evolved into more horizontal, multi-stakeholder public-private partnerships (PPPs) (38,39). One important reason is that the multi-stakeholder PPP model generates the necessary common ground where governmental bodies, universities, patient organizations, health foundations, and the private sector can combine resources and expertise. PPPs allow these stakeholders to address jointly the grand challenges within the field of pharmaceutical innovation that are of mutual interest. In simple terms, PPPs have the potential to make things happen that would not be possible in their absence (40–42).

Van Ham and Koppenjan defined multi-stakeholder PPPs as “*the cooperation of some sort of durability between public and private actors in which they jointly develop products and services and share risks, costs and resources which are connected with these products*” (43). Within the area of pharmaceutical R&D, there are essentially two main types of multi-stakeholder collaborative research initiatives or PPPs (44):

- Product Development PPPs, which have positioned themselves to address the global prosperity gap by developing pharmaceutical solutions (vaccine, diagnostic, drug) for low and middle income countries with an alternative (not-for-profit) business model (45–47).
- Precompetitive PPPs, which aim to generate novel scientific concepts (e.g. disease targets and research models) and infrastructures (e.g. databases) through effective collaboration between multiple public and private entities based on mutual trust, pooling of complementary expertise and knowledge, and sharing of rewards. Potential disputes over issues such as intellectual property are avoided by limiting activities to the precompetitive space (48).

Also important, and closely related to these two types of PPPs, is the group of Access PPPs. These aim to improve accessibility to a specific treatment modality through mass-drug administration programs, in order to alleviate disease burden in low and middle income countries and to overcome obstacles in the distribution system of treatments (49). An interesting initiative to increase patient access to expensive biologics in low and middle income countries is the joint development of affordable, high quality biosimilars by a consortium of companies from low and middle income countries. This development is undertaken in collaboration with academic partners and coordinated by the Utrecht Centre for Affordable Biotherapeutics.

Table 2 shows examples of precompetitive and product development PPPs.

**Table 2:** Examples of Precompetitive and Product Development PPPs

	Mission	Ref.
Precompetitive		
Critical Path Institute	To foster development of new evaluation tools and standards for drug therapy trials, which accelerates regulatory qualification and medical product approval and adoption.	(50,51)
Innovative Medicines Initiative	To improve health by speeding up the development of, and patient access to, innovative medicines, particularly in areas where there is an unmet medical or social need.	(52,53)
Top Institute Pharma	To establish, support and manage public-private collaborations between academia and the (inter-) national pharmaceutical industry to create ‘health & wealth’.	(54)
Structural Genomics Initiative	To catalyze research in new areas of human biology and drug discovery research by focusing on less-well-studied domains of the human genome.	(55,56)
Product development		
Medicines for Malaria Venture	To reduce the burden of malaria in disease-endemic countries by discovering, developing and facilitating delivery of new, effective and affordable antimalarial drugs.	(57)
Drugs for Neglected Diseases initiative	To develop new drugs or new formulations of existing drugs for people living with neglected diseases.	(58)
International AIDS Vaccine Initiative	To ensure the development of safe, effective, accessible, preventive HIV vaccines for use throughout the world.	(59)
Pediatric Praziquantel Consortium	To develop, register and provide access to a suitable pediatric praziquantel formulation for treating schistosomiasis in preschool-age children.	(60)

According to Lim, the average number of multi-stakeholder PPPs launched per year has grown from 8 in 2001–2003 to 54 in 2011–2013 (38). A major contributor to this growth has been the launch of various high-profile precompetitive public-private initiatives, involving multiple consortia, such as the European Innovative Medicines Initiative (IMI), the Dutch Top Institute Pharma, and the US Foundation for National Institutes of Health. Faster Cures has included in its consortiapedia catalogue more than 350 consortia profiles, both public-public as well as public-private (38).

The literature clearly indicates that the concept of PPP has matured. A non-exhausting bibliometric analysis performed through PubMed (searching by “Public-Private Sector Partnerships”[Mesh] OR “public-private partnership”[TIAB] OR “public-private partnerships”[TIAB]) revealed over 2000 citations, of which more than 300 could be directly linked to the concept of multi-stakeholder collaborative research. Closer evaluation reveals that as successfully launched PPPs grew over the last decade, the number of citations on the concept of “biomedical R&D” PPP also increased, from around 35 between 2001–2005 to around 225 in 2011–2015.

Such a high number of citations demonstrates that the concept of PPP has been adopted fully by funders, academia, and the private sector as an important tool that unites two intrinsically different systems of knowledge creation. Within academia, the emphasis is on curiosity-driven, conceptual, publicly accessible research with a long horizon. Within industry, competitive advantage is pursued by shielding in-house research findings and disclosure through patents (61). This intrinsic difference was once considered as being the core of the obstacles to successful public-private collaboration in biomedical R&D (62–64). Organizational aspects of boundary-spanning activities between universities and industry (62), such as conflicts over IP and university administration (64), have also been mentioned by industry as an important barrier towards successful collaboration with universities (65,66). It appears that PPPs in the precompetitive stage, where scientific concepts are developed, offer a perfect level playing field for all stakeholders to facilitate translational medicine, and ultimately to improve pharmaceutical R&D productivity.

## What are the Main Drivers for key Stakeholders to Embrace the Multi-Stakeholder PPP Model?

As former NIH director Elias Zerhouni put it, "*The way forward in multidisciplinary research is to engage in predictive, personalized, pre-emptive and participatory medicine. For the creation of the optimal innovation climate that would allow for such a strategy, public-private partnerships have been proposed*.” (67). We fully agree with this statement, but full exploitation of basic science into clinical utility also requires more. It needs the lifting of existing boundaries between scientific disciplines and stakeholders, resulting from traditions, policies, and bureaucracies (68). Apart from an increased level of “transdisciplinarity”, exploitation depends on integrating the contributions of the major stakeholders involved in translational medicine: academia, governmental bodies, small and large bio-pharmaceutical industry, health foundations, and patient organizations. The successful integration of resources and expertise from multiple stakeholders, especially between universities and industry, is built on a clear understanding and alignment of their missions and needs within biomedical R&D. Furthermore, public and private stakeholders play different roles in the biomedical research translation continuum (3), so an awareness of the intrinsic motivation of the various stakeholders seeking a multi-stakeholder PPP collaborative model will help to value properly public-private collaboration.

## Universities

Public organizations, mainly universities, focus primarily on technology *push*. Their footprint can be seen at all stages of the research and development continuum. They are the major providers of basic science concepts, and universities, especially in the US and the EU, now consider commercialization of their research activities an integral part of their mission, as mentioned above. Although commercialization has been identified as a reason for universities to engage with industry (36,69), academics in particular are driven by a number of motivators: 1) the opportunity to learn about industry challenges and research activities, with feedback on applicability of research and the chance to become part of a network; 2) access to in-kind resources (materials, research expertise and equipment); and 3) access to alternative sources of public and private funding.

The term “technology” encompasses, among other things, biochemical findings and new molecular entities, implying an innovation which is pushed through R&D to the market. Here, academic research groups act based on curiosity and on their passion for performing hypothesis-driven research. Crowley mentions the intrinsic level of unpredictability as an important and also attractive characteristic of curiosity-driven research (70). There are various examples of important solutions to clinical problems that were derived from basic science that could never have been predicted at their outset, such as the successful treatment of leukemia with Bcr-Abl tyrosine kinase inhibitors and the clinical use of bisphosphonates (70).

## Pharmaceutical Industry

Private entities, mainly from the industrial side, focus on market *pull*. They react to the needs of patients through market analysis, and have the appropriate resources, knowledge and capabilities at their disposal to bring new, promising and often innovative therapies to the market. As discussed above, the pharmaceutical industry has increasingly embraced different models of open innovation, including PPPs (25), because of the need to target diseases of greater complexity, and because they are faced with a high attrition rate in drug development. Various scholars (63,71,72) have listed key incentives for industry seeking collaboration with universities. These include gaining access to basic knowledge; improving the ability to solve problems; gaining access to new tools and techniques for the development of new technologies; improving a firm’s reputation in the labor market and among potential partners; entering into the academic network; and exploiting opportunities for public funding.

Several studies and comments have pointed out that funding of clinical research by the pharmaceutical industry is strongly associated with pro-industry results (73–76). We do not take this concern about the influence of the pharmaceutical industry on the development and use of medicines lightly. However, we also believe that influence in the basic science stage, which is by its nature precompetitive and concept-driven, will be limited for several reasons. First, using data from the Carnegie Mellon Survey on industrial R&D, and investigating over 30 manufacturing industries, Cohen *et al*. showed that the pharmaceutical industry is unique and highly dependent on basic science (77). The latter has been confirmed by other studies (78). Second, as summarized by Perkmann (71), there are several studies showing that university-industry collaboration doesn’t negatively impact university’s research productivity, nor does it result in shifting away from basic science and more towards applied research. Finally and perhaps most importantly, as mentioned above, the intrinsic motivation of academics to engage with industry goes beyond mere financial gain (36). This is especially true in a PPP setting, in which funding of public partners and SMEs is in general provided by governmental bodies and/or health foundations (52,79). Taking the various positions of both public and private entities into consideration reveals that they are really interdependent (80). As stated by Crowley (70), “*Academia and industry need each other to effect substantive improvements in health. Without substantive collaborations between the two, maximum advances in healthcare for the US public are unlikely*”.

## Health Foundations

Apart from universities and industry, other stakeholders have also started to embrace this type of partnership to reach their objectives. Health foundations or charities increasingly demonstrate that PPPs can be instrumental in obtaining maximum benefit for the patients they serve (79). In reviewing their research portfolio, these organizations could see that there was considerable progress towards advancing basic disease understanding. However, it was also observed that translation of this knowledge into clinical proof of concept was lagging considerably, with a need for alternative strategies. Of course, health foundations do not possess the budgets of larger pharmaceutical companies or governmental funders (81,82), but, based on a survey amongst 11 major US health foundations, Chang identified a number of non-monetary value drivers that health foundations consider of added value in a partnership (83). First, health foundations can provide the necessary clinical network and access to patients. Second, because of their central position, foundations have relationships with and unfettered access to the world’s leading academic experts who can facilitate understanding of the basic science, but who can also play a role in clinical trial design and evaluation. Third, as already mentioned, foundations can help to attract additional funding to a partnership, and place great emphasis on collaboration and information sharing. Finally, although knowledge sharing amongst partners in a PPP can be cumbersome due to concerns about IP/trade secrets and/or other adverse competitive consequences, health foundations have demonstrated that they can act as a trusted intermediary or broker. They can ensure proper storage, handling, analysis and/or mediated revealing of information (e.g. aggregation and anonymization of information before it is distributed within the PPP).

## Patient Organizations

Numerous health foundations, such as the Cystic Fibrosis Foundation (84) and the Polycystic Kidney Foundation (85), were originally founded by patients (or their carers) with a key objective: “*to find treatments and a cure”* for a specific disease or group of diseases (86,87). A review of 75 Innovative Medicines Initiative (IMI) projects reveals that European or international patient associations (such as EURORDIS, the European Patient Forum and the International Alliance of Patients’ Organizations) are participating in a total of 16 IMI PPPs (88). Although not all 16 projects provide detail on organizations’ roles in the work program, closer examination reveals that patient participation occurs at three levels (89). The lowest level of participation is to support partnerships with the dissemination of project results to patients and public. Examples in this respect are the IMI projects EPAD (90) and PharmaCog (91) projects. The next level of patient participation, best exemplified by GetReal (92), Protect (93), and U-Biopred (94), is to ensure that the perspective of patients and patients’ organizations is included from the start of the work program via focus groups, patient input platforms, and memberships to other project governance bodies. Finally, EUPATI is a prime example of the highest level of patient participation, a patient-led project. It focused on training close to 100 patients or their representatives in all aspects of medicines development, and on developing an extensive, multi-language training toolbox to be rolled out across Europe (95).

## Regulatory Agencies

Finally, regulatory and health technology assessment agencies, such as the EMA and the Dutch Medicines Evaluation Board, also see the PPP model as a platform that allows them to share data, expertise, and resources, and to enhance their understanding of other stakeholders’ perspectives (50,51,96–98). Nowadays, these public bodies participate in a variety of PPPs, including those focusing on the development of new evaluation tools and standards (Adapt-SMART, C-Path, Escher (97–100)); training programs in drug regulatory sciences e.g. PharmaTrain (101); and new approaches for incorporating real life data into drug development (e.g. GetReal (92)).

## How to Evaluate the Performance & Impact of Precompetitive Research PPPs

With so many PPPs launched in the last two decades, we agree with Freire that it is time to “*move beyond the buzzword*” of PPPs as engines that simply facilitate the translation of basic fundamental research, looking also at their impact on proven clinical application and eventually, medical practice (102). Like biomedical research in general, there is a growing demand to better understand the socio-economic value of biomedical R&D PPPs (103,104). Apart from understanding socio-economic value to ensure continued funding support, other identified reasons for this move towards evaluating the (research) impact of PPPs include the requirement for accountability. Research organizations need to judge and manage their performance and to improve understanding of which research (model) ultimately leads to the desired impact (103).

In the last decade, various scholars have independently investigated indicators to evaluate the performance of research PPPs (2,105) and of university-industry alliances in general (62,63,106). As depicted in Table 3, the proposed frameworks for a performance measurement system are comparable, and follow the basics of the logical framework method. This involves defining objectives and securing the necessary inputs through to producing a concrete output and eventually generating the desired impact (107). The adoption of unambiguous definitions for each stage, generally accepted by all stakeholders, will be essential, because this ensures a clear distinction between input, process, output, short term (or intermediate) outcomes and long term outcomes/impact. Building on existing literature (2,62,108,109), we propose the definitions that follow below as a starting point for further discussion.

**Table 3:** Proposed logical frameworks, including key stages, as a PPP performance measurement system

Logical framework key stages						Reference
-	Input	In-process	Output	Impact		Perkmann *et al*. (62)
-	Input	Process	Output	Outcome		Denee *et al*. (2)
Stated objectives	Inputs	-	Mediators^#^	Intermediate outcomes	Final outcomes	Innovative Medicines Initiative (105)

^#^Resources, processes and facilities that can link inputs to outcomes

The input stage revolves around “*the PPP’s ability to bring together human, financial and physical resources, public and private researchers’ capabilities and motivation, and the partners’ knowledge and experience*”. These inputs provide the basis for the following process stage, which reflects “*the PPP’s ability to pursue a high-quality research program, as well as educational and/or other services that are relevant to all stakeholders, and to create an environment of genuine trust that allows ample sharing of knowledge and resources throughout the PPP*”. The process stage will result in the generation of various concrete outputs or “*the PPP’s ability to generate immediate tangible scientific knowledge, products and/or services”.* Depending on “*the PPP’s ability to encourage their use in the short term*”, these outputs will translate into short term (or intermediate) outcomes. Finally, long term outcome/impact can be defined as “*the longer term economical and health impact of the PPP’s generated output, for society, patient as well as the stakeholders involved*”.

As depicted in Table 4, a number of performance indicators have been suggested for each stage of the proposed logical framework, to allow the implementation of PPP performance measurement in practice (2,62,105). A number of indicators are also included for biomedical research output and outcome that are appropriate for research PPPs (110). Finally, building on top of work by Denee *et al*. and Thonon *et al*. (2,110), we suggest classifying the indicators into five categories or domains: Networks and collaboration; research activity and knowledge; knowledge sharing and dissemination; human capital; and financials and operations.

**Table 4:**
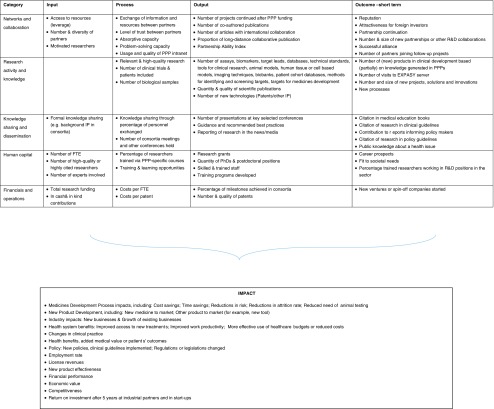
Reported performance indicators to be considered in a research PPP performance measurement system (2,62,105,109,110), classified into 5 categories derived from Denee *et al*. (2) and Thonon *et al*. (110)

Establishing a framework, a clear definition of the key stages, and a set of performance indicators provides a good starting point for implementation of a meaningful PPP performance measurement system. However, several challenges, especially around outcomes and long-term impact, have been reported that will need to be taken into consideration to ensure that a PPP performance measurement system is implemented that is endorsed by all the stakeholders involved.

To start with, the lengthy timeline associated with pharmaceutical R&D, combined with research becoming more and more multidisciplinary in nature (and consequently involving many different processes, individuals, and organizations), makes it challenging to attribute directly a specific contribution from a group or PPP to a specific health or socio-economic impact (103,111). On top of this, ‘knowledge creep’ is considered a challenge when assessing the impact of research, because policy makers have a tendency to respond slowly to translating accumulating evidence into policy and/or guidelines, and often do so without recognition of the contributing research (112).

In line with Penfield *et al*. (103), given that the type of impact stakeholders anticipate varies according to the type and scope of a PPP, impact-specific challenges may prohibit a fair comparison of impact between PPPs. Linking the results of a clinical trial (output) to clinical benefit (outcome) will obviously be more straightforward than discovery of a new drug candidate or a specific training program to clinical benefit (1). A recent systematic review by Thonon *et al*. revealed a total of 57 indicators to measure output and outcome of medical research. Most identified indicators were in fact the many different indexes (e.g. h-index) currently available to measure research production and impact. With research being complex, non-linear, and unpredictable in nature (113), there is a propensity to ‘count what can be easily measured’ (114,115), rather than ‘measuring what counts’ in terms of significant, enduring changes (114–116). We concur with this, however the underlying problem is not the availability of possible indicators, but rather the measurability of indicators, especially outcome ones. A series of expert workshops, using IMI and TI Pharma as examples, revealed that, with regard to output, 9 out of a total of 10 suggested indicators were considered measurable; however, with regard to outcome/impact, only 2 out of a total of 12 suggested indicators were considered measurable (109). This difficulty of measuring outcome, especially in disciplines such as social and policy science, was also reported in a recent analysis of 162 impact case studies from the 2014 UK Research Excellence (117). As highlighted by Perkmann *et al*., the intangibility of many outcome indicators will require the identification of suitable and measurable proxies (62). This is especially true when it comes to multi-stakeholder public-private collaborations, for which various soft elements of critical success factors have been identified, such as trust (118), value recognition (119,120), communication (34,119,121), and stakeholder involvement (119–122).

Research impact assessment studies are all part of a relatively new scientific endeavor. With PPPs becoming increasingly multidisciplinary, involving multiple stakeholders with competing interests and generating a multidirectional exchange of knowledge, a recent review of the normative literature on infrastructural PPPs suggests that the logic model of impact assessment (cf. Table 3) doesn’t fully capture the interaction between researchers, funders, innovation intermediaries and end-users (123). Currently, other models have been developed and are under evaluation, which take into consideration the multiple-stakeholder complexity mentioned above (124). Several scholars have started to argue that in the end, research impact assessment is most likely to be assessed through a multi-indicator, multi-method approach (113,114) involving a mix of quantitative analysis (e.g. publications, citations, patents); interviews with key stakeholders; peer assessment; case studies; and ultimately routine engagement with the end-users of research.

## Precompetitive Biomedical PPPs Starting to Generate Tangible Outcomes

A quick glance of the scientific literature reveals a number of reviews highlighting that considerable outputs, such as publications, patent applications, tools, in silico and animal models, training modules, and open-source databases have been generated by the various biomedical R&D PPPs initiated in the last two decades (40,42,55,97,125–127). However, as mentioned above, the true success of the precompetitive multi-stakeholder public-private collaboration model ultimately goes *beyond* mere outputs. Success is about its ability to generate tangible outcomes, and in the long run a sustainable socio-economic impact. Questions that must be addressed include: Which research has translated into novel disease targets, or in policy and/or guideline changes? To what extent have developed tools and models been adopted by relevant stakeholders? What is the number of end-users of the various infrastructures and databases that have been generated? How many PPPs continue beyond their original term? In contrast to the increasing number of publications on the concept of biomedical R&D PPPs mentioned above, publications focusing specifically on (short-term) outcomes and impact of biomedical R&D PPPs supported by tangible evidence are still limited.

## Case Studies

In the following sections four case studies differing in size, geographical spread and research focus will be discussed.

### The Structural Genomics Consortium (SGC)

In 2014, RAND Europe and the Institute on Governance evaluated the SGC model of operation (56). SGC is a pre-competitive public-private partnership that was founded in 2003, and operates from six academic institutions - the University of Toronto (Canada); the University of Oxford (UK); Karolinska Institute (Sweden); University of North Carolina (US); the State University of Campinas (Brazil); and the Goethe University Frankfurt (Germany). The partnership comprises an open collaborative network of scientists in hundreds of universities around the world and in nine global pharmaceutical companies. Its main goal is to accelerate “*research in human biology and drug discovery by making all of its research output freely available to the scientific community*” (128). The focus is on providing a boost to drug discovery by determining 3D protein structures in biomedically relevant areas in a cost-effective manner. Apart from perspectives on the approach, strengths and weaknesses of the model and lessons learnt, part of the evaluation was to extract the value of knowledge in line with the framework presented above: outputs, outcomes and impacts of the SGC. In 2013, the SGC had produced a total of 1195 protein structures and 83 sequences; 17 chemical probes and compound tools; and 98 antibodies. It made these publicly accessible through established databases (Protein Data Bank, Uniprot). More than 1400 clones were provided to both the private and academic sectors. This considerable output was accompanied by 452 peer-reviewed publications, including Nature, PNAS and PLoS One, and by attendance and presentations at well over 250 conferences. With regard to outcomes, defined as “*the utility of those outputs in delivering additional impacts*”, the report focused on three aspects: the reach of the SGC research, the influence of SGC researchers and the economic impact of SGC developments (56). Using the list of indicators and categories depicted in Table 4 as a point of reference, the reach of the SGC was identified in the area of *human capital* (collaboration with over 500 scientists and training of over 300 scholars). Its influence was acknowledged in the areas of *human capital* (over 100 staff moved to academia, industry and business schools) and *knowledge sharing & dissemination* (around 100 meetings with industry, policy maker discussion and workshops). Assessing the economic impact of the SGC, such as the costs of new products or valuation of new companies, was difficult. Apart from a $15 m financing of spin-out Tensha Therapeutics, a second spin-out (1DegreeBio) estimated a potential $1bn cost-savings in R&D expenditure relating to the identification of effective antibodies (56). Finally, based on several assumptions, the revenue of products related to the SGC was appraised at a value of over $60 m CAD. Important to note is that apart from these tangible deliverables, more intangible ones were also mentioned. As mentioned by private sector representatives in interviews, the SGC also provided a platform that allowed pooling of resources and sharing of expertise, saving costs on bureaucracy and generating a more efficient research processes.

### The Innovative Medicines Initiative (IMI)

The difficulty to attribute scientific outcomes to input mentioned earlier was confirmed by the SGC evaluators as a key limitation for a robust analysis. Research is not performed in splendid isolation, but is part of a complex network of interactions that contribute jointly to a specific scientific breakthrough (129). This complexity, and also the existing lengthy timelines for pharmaceutical R&D (5), was also mentioned in a recent report on the socio-economic impact of the IMI (105). With the aim of improving the competitive situation of the European Union in the field of pharmaceutical research, the European Commission (EC) and the European Federation of Pharmaceutical Industries and Associations (EFPIA) implemented the IMI in 2008 (52,98). With a total budget of over EUR 5 billion (IMI1: 2 billion in 2008–2013; IMI2**:** 3.3 billion in 2014–2024), IMI is generally considered the largest biomedical PPP in the world. It aims to support the development of next generation vaccines, medicines and treatments, focusing on, among others, the improvement of the current drug development process and reduction of the time to reach clinical proof of concept. Currently, IMI has funded over 80 public-private consortia spanning the complete drug development cycle, including training, post-marketing and regulatory science.

In 2016, the IMI appointed an expert group to assess the socio-economic impact of the first nine IMI projects (total value >EUR 200 million; completed early 2016) of which eight were focused on precompetitive research and one on training. Although in the report the complexity of interactions between various measures of inputs, outputs, outcomes and impact was acknowledged, the expert group chose to adhere to the logical framework (See Table 3) to assess the performance of the project. In terms of output, the nine projects combined generated over 550 scientific publications (proxy for research activity and knowledge sharing); cell-based or animal models in five different (disease) areas; three biobanks (diabetes, medicine-induced injuries, pain); novel imaging techniques in the area of diabetes; new or improved biomarkers; a patient cohort database; and novel tools to test biomarkers. In addition, a plethora of novel targets, processes, approaches, and methods were reported that could incite ongoing and new collaborative research. Tangible outcomes that were mentioned in the report were four start-ups/spin-offs; patents; several assay products; tools and animal models being commercialized; and a training program.

Taking into consideration the complexity of pharmaceutical R&D and its lengthy timelines, the expert group considered tangible socio-economic impacts, such as delivery of novel treatments; cost and time savings; reductions in risk and attrition rate; and the reduced need for animal testing beyond the scope of the IMI. The impact IMI projects can generate is primarily related to improvements and amendments within the medicines development process itself, also defined by the expert group as the “*pathway to socio-economic impact*” (105). However, generating true tangible socio-economic impact will rely heavily on recognition of the benefits arising from the IMI, and subsequent actions by pharmaceutical companies, regulators, payers and policy makers through adoption of novel processes, the implementation of novel or updated guidelines and policies, and so on.

### The Dutch top Institute Pharma (TI Pharma)

This PPP (total budget: EUR 274 million) ran between 2006 and 2014 (motto: ‘health and wealth’) and built pharmaceutical research and development networks in five disease areas: (auto)immune diseases, cardio-vascular diseases, cancer, infectious diseases, and brain diseases. Enabling technologies included therapeutic target finding; validation & animal models; lead selection & in-silico modelling; predictive drug disposition & toxicology; biomarkers & bio-sensoring; and drug formulation, delivery & targeting. In addition to contributions by Dutch universities, Dutch SMEs and international big pharma companies, the Dutch government supported this PPP with a total contribution of EUR 135 million. TI Pharma operated as an independent body, and the only condition for the Dutch government’s contribution was that projects were in line with the recommendations from the ‘WHO Priority Medicines report’ published in 2004 and (updated in 2013 (130)). A total of 26 academic institutions, 43 SMEs, and 20 big pharma companies were partnering via 74 TI Pharma projects. At the end of the 2013, TI Pharma’s output consisted of 470 trained PhD and post-doctoral fellows; almost 750 publications; 41 lead compounds, lead series and libraries; 18 novel formulations; 11 biomarkers; 33 preclinical models; 28 clinical models; 11 research databases; and 87 research tools (assays, discovery models) (54). Several tangible early outcomes were reported. Regarding *human capital,* of the 257 TI Pharma fellows who had finished their project by the end of 2013, an impressive 98% continued their career in industry, academia and other pharma (e.g., regulatory) and non-pharma organizations. In the area of *research activity and knowledge,* clear benefit for patients is anticipated through the delivery of a safe morphine dosage regimen for newborns; new clinical protocols for COPD; a vaccine candidate against acute myeloid leukemia; and a disease registry for rare metabolic diseases, which will facilitate development of diagnosis and treatment for this specific group of diseases. A total of 74 follow-up projects could also be identified, which built on the outputs of various TI Pharma projects (*Networks & collaboration)*. Finally, in 2015 TI Pharma merged with another Dutch PPP, resulting in Lygature and continues to successfully manage international PPP projects (current portfolio: >15 projects).

### The Alzheimer’s Disease Neuroimaging Initiative (ADNI)

One of the most well-described precompetitive biomedical R&D PPPs is the Alzheimer’s Disease Neuroimaging Initiative or ADNI (131–137). ADNI was initiated in 2004, and with a total budget of more than $150 million is considered the biggest PPP in Alzheimer disease (AD) research. Alzheimer disease is a complex disorder affecting tens of millions of people around the world (138), and translation of disease understanding into viable treatment options remains disappointing (139). Apart from its intrinsic complexity, one major limitation faced by both academic and pharmaceutical partners during clinical trials was that outcome measures were limited to clinical and cognitive ones. Through ADNI, US leading Alzheimer research centers, the National Institute on Aging, 13 pharmaceutical companies and two not-for-profit foundations all combined resources and expertise to facilitate development of effective AD treatments, by developing, validating, and qualifying a novel set of imaging and other biomarkers for clinical trials (132,133). Originally planned as a five-year project (ADNI-1), with an important coordinating effort by the Foundation for the National Institutes of Health, the partnership has been successful in: 1) attracting additional funding to extend (ADNI-GO) and renew (ADNI-2 & ADNI-3) the overall project until mid-2021; 2) increasing the number of partners to more than 30; and 3) attracting the Canadian Institutes for Health Research to join (133). To date, there have been no successful clinical trials with AD-modifying drugs. However, Weiner *et al*. and Jones-Davis and Buckholtz recently summarized the key outputs and outcomes of ADNI, which in essence started out as a multi-site, longitudinal study of normal cognitive aging, mild cognitive impairment, and early AD (132,133).

All data (PET, MRI, clinical, biospecimens, genetics) acquired during the ADNI project to date is housed in a database hosted by the Laboratory of Neuroimaging, and is open to the entire scientific research community. This has resulted in over 14 million downloads, and more than 750 publications citing the use of ADNI data (132,140), of which a number are in high-impact journals, such as Nature, PNAS, and PLOS One (141). Populating the database continues, with the upload of data derived from the use of ADNI samples. Apart from ensuring its own sustainability, ADNI has also looked beyond the US border (132). It has been a key driver in promoting the ADNI concept worldwide, resulting in ADNI projects based in North America, Europe, Japan, Australia, Korea, and Argentina, which are united through Worldwide-ADNI (142). The ADNI concept has also inspired other initiatives focusing on closely related aspects of AD (e.g., traumatic brain injury as an AD risk factor) and other PPPs (e.g., developing biomarkers for Parkinson’s disease).

By making data and samples available, the infrastructures and databases described above have also made an important contribution to enhancing the basic understanding of various complex diseases. As well as providing open-access to data and sharing of samples, ADNI has also made available a set of protocols and methods related to the study itself, to MRI and PET analysis and acquisition, and to biomarker and proteomic analysis. This allows comparison of data gathered across multiple sites. According to Weiner *et al*. “*pharmaceutical companies developing disease-modifying treatments for AD and studies funded by the National Institutes of Health and private foundations have used ADNI methods in virtually all their clinical trials*” (132). The knowledge of the progression of AD pathology, of biomarker interrelationship, and of genetic risk factors for the disease that ADNI has generated, is also being acknowledged in recently issued draft regulatory guidelines on clinical investigation of medicines for the treatment of AD and other dementias (143,144). In addition, to helping enrich recruitment in regulatory clinical trials for Mild and Moderate AD, the first two biomarkers (CSF Aβ and tau protein; and low hippocampal volume) have been or are in the process of being qualified by the EMA and FDA (145,146).

## Concluding Remarks

Despite the intrinsic difficulties involved in measuring the impact of biomedical PPPs, recent evaluations of the SGC, IMI, TI Pharma and ADNI highlighted in this review provide clear evidence that precompetitive biomedical PPPs have started to generate tangible outcomes. These outcomes are in areas where not only the pharmaceutical industry and academia, but also health foundations, patient organizations and regulatory agencies share a mutual interest. These include: 1) providing the necessary infrastructure and databases to facilitate the discovery of novel disease targets and leads; 2) increasing basic understanding of complex diseases, including novel models and standards; 3) capacity building through jointly designed training programs; and 4) providing a platform to allow exchange of different perspectives on, for example, the regulatory system and regulatory science (38,44,147)*.* Considering the long timelines for uptake of validated new developments in the biomedical/pharmaceutical field, continuation of PPPs beyond their original time span (often 4–6 years) will certainly contribute to PPPs reaching their full potential. This can be achieved either through a continuation of funding (ADNI, IMI) or by changing the business model (TI Pharma, IMI-Pharmatrain).

As highlighted above, pharmaceutical R&D is complex and characterized by lengthy timelines, and one could easily argue that the challenges mentioned in this review make it very difficult to properly assess the long-term impact of the multi-stakeholder PPP concept on medical practice. However, we should continue to improve the metrics. Given the growing number of PPPs, the availability of funding and resources will become a limiting factor, and will “force” stakeholders to become even more prudent in selecting which PPP initiatives to back or join, and which ones to avoid (1). In simple terms, partners, funders, and civil society will increasingly seek confirmation of the incremental value achieved through partnerships.

This paper highlights a link between the emergence of the PPP concept and the growing ‘popularity’ of open innovation in the biomedical/pharmaceutical R&D world. One may wonder how this open innovation model will evolve, and both whether and how the format of PPPs will change with it. We have already seen changes since the time when a typical PPP was based on an academic and industrial pillar, with governmental or other third party funding as ‘carrot’. Over time, health foundations, patient organizations and regulatory scientists have regularly joined in (79,85,94,96). So, what is next? More funding and ‘research priority setting’ from philanthropists, also coined as philanthrocapitalism (148)? The future will tell.

## Abbreviations

AD: Alzheimer’s disease
ADNI: Alzheimer’s disease neuroimaging initiative
EMA: European medicines agency
FDA: Food and drug administration
IMI: European innovative medicines initiative
PPP: Public private partnerships
R&D: Research & development
SGC: Structural genomics consortium
SME: Small and medium-sized enterprise
TI Pharma: Top institute pharma
WHO: World health organization
